# The Bacterial and Fungal Gut Microbiota of the Greater Wax Moth, *Galleria mellonella* L. Consuming Polyethylene and Polystyrene

**DOI:** 10.3389/fmicb.2022.918861

**Published:** 2022-07-05

**Authors:** Juliana M. Ruiz Barrionuevo, Brayan Vilanova-Cuevas, Analía Alvarez, Eduardo Martín, Agustina Malizia, Alberto Galindo-Cardona, Ricardo E. de Cristóbal, M. Angelica Occhionero, Adriana Chalup, A. Carolina Monmany-Garzia, Filipa Godoy-Vitorino

**Affiliations:** ^1^Instituto de Ecología Regional (IER), Universidad Nacional de Tucumán (UNT)–Consejo Nacional de Investigaciones Científicas y Técnicas (CONICET), Tucumán, Argentina; ^2^Facultad de Ciencias Naturales e Instituto Miguel Lillo, Universidad Nacional de Tucumán (UNT), Tucumán, Argentina; ^3^Department of Microbiology and Medical Zoology, School of Medicine, University of Puerto Rico, Medical Sciences Campus, San Juan, Puerto Rico; ^4^Planta Piloto de Procesos Industriales Microbiológicos (PROIMI-CONICET), Tucumán, Argentina; ^5^Fundación Miguel Lillo (FML), Tucumán, Argentina; ^6^Centro Científico Tecnológico (CCT-NOA SUR), Consejo Nacional de Investigaciones Científicas y Técnicas (CONICET), Tucumán, Argentina; ^7^INSIBIO (CONICET - UNT), Instituto de Química Biológica “Dr. Bernabé Bloj”, Facultad de Bioquímica, Química y Farmacia, Universidad Nacional de Tucumán, Tucumán, Argentina

**Keywords:** plastic pollution, insect gut, plastivore, Argentina, bacteria, fungi

## Abstract

Plastic production has been increasing exponentially in the last 60 years, but plastic disposal is out of control, resulting in the pollution of all ecosystems on Earth. Finding alternative environmentally sustainable choices, such as biodegradation by insects and their associated gut microbiota, is crucial, however we have only begun to characterize these ecosystems. Some bacteria and one fungus have been previously identified in the gut of Greater Wax Moth larvae (*Galleria mellonella* L., Lepidoptera, Pyralidae) located mainly in the Northern hemisphere. The aim of this study was to describe changes in the gut microbiota associated with the consumption of polyethylene and polystyrene by the Greater Wax Moth in Argentina, considering both bacteria and fungi. Larvae were fed polyethylene, polystyrene and beeswax as control for 7 days. Next generation sequencing revealed changes in the bacterial gut microbiome of the wax moth larvae at the phyla and genus levels, with an increase in two *Pseudomonas* strains. The fungal communities showed no differences in composition between diets, only changing in relative abundance. This is the first report of both bacterial and fungal communities associated with a plastivore insect. The results are promising and call for more studies concerning a potential multi-kingdom synergy in the plastic biodegradation process.

## Introduction

The massive production of plastic started only 50–60 years ago (i.e., 1960) and recent quantifications estimate 360 million metric tons (Mt) in 2018, with an average annual growth rate of 8.6% (1.7 million tons; PlasticsEurope, 2019). Most items used by humans are made of this inexpensive, flexible and long-lasting material. Its’ unregulated production, use, extremely rapid disposal, and low rates of recycling have resulted, though, in plastic waste accumulation in every biome on Earth, including marine, freshwater, and terrestrial habitats (Rochman, 2018; Bucci et al., 2019; Malizia and Monmany-Garzia, 2019). Next to climate change, it is fast becoming the most important environmental issue of our time. Due to the multiple effects of plastic pollution on the health of ecosystems, solutions toward mitigating these consequences are being sought at different levels of socio-ecological organization (Rochman, 2018; Przemieniecki et al., 2019).

Recently explored solutions for plastic pollution mitigation include plastic biodegradation. Microplastics (i.e., <5 mm) represent a new substrate for distinct microbial colonization and biofilm formation, called the “Plastisphere” (Zettler et al., 2013; McCormick et al., 2014), suggesting that many of these organisms are able to degrade the material. Biodegradation effects on ubiquitous polyolefins such as polyethylene (PE) and polystyrene (PS), may be exerted, in fact, by bacteria, and fungi (or associated enzymes) isolated from the open environment and from the guts of insects (Yang et al., 2015a,b; Bombelli et al., 2017; Brandon et al., 2018; Wang et al., 2020; Woo et al., 2020) and other animals (Leonov and Tiunov, 2020; Song et al., 2020). Concerning insect gut microbiota, a few studies showed evidence of plastic biodegradation mostly by bacteria associated with coleopteran and moth species. For example, *Plodia interpunctella* (Lepidoptera, Pyralidae) degraded polyethylene by the action of at least two gut bacteria: *Enterobacter asburiae* and *Bacillus* sp. (Yang et al., 2014). When examined at the community level, the gut microbiota of insects fed with plastic diets showed changes in structure and composition, as indicated by the relative abundance and identity of the species, respectively (Przemieniecki et al., 2019; Cassone et al., 2020; Lou et al., 2020). For instance, Przemieniecki et al. (2019) reported that gut bacterial communities of *Tenebrio molitor* larvae fed with oat and cellulose diets, had similar composition, while those individuals fed with polyethylene formed another taxonomic group, and those with a polystyrene diet had the highest dissimilarity in composition.

Some gut bacteria of the Greater Wax Moth, *Galleria mellonella* (Lepidoptera), a voracious ‘plastivore’, consuming and degrading polyethylene (Bombelli et al., 2017; LeMoine et al., 2020) and polystyrene (Lou et al., 2020), have been identified. High abundances of the bacteria *Bacillus* spp., *Serratia* spp., *Acinetobacter* spp., *Enterobacter* sp. D1, *Enterococcus* sp., and *Massilia* sp. have been recorded in individuals consuming these materials (Ren et al., 2019; Cassone et al., 2020; Lou et al., 2020; Jiang et al., 2021). The phyla Firmicutes and Proteobacteria have been pointed out as part of the core gut bacteria of the Greater Wax Moth, given their predominance in the guts of larvae under nine different feeding conditions (Lou et al., 2020). Most studies examining these changes of gut microbiota at the community level only considered bacteria, while the analysis of fungal species and their co-occurrence with bacteria have been largely unexplored. Only one *G. mellonella*’s gut fungus, *Aspergillus flavus*, has been reported participating in the degradation of polyethylene (Zhang et al., 2020). But, the identity of the wax moth’s core gut fungi remains unknown. The combined processes of gut bacteria and fungi have been suggested as an opportunity for insects to deal with challenging diets (Mason et al., 2019). Given the important role of fungus in plastic biodegradation (Gautam et al., 2007; Shah et al., 2008), we need to include fungal communities in the description of *G. mellonella*’s gut microbiome consuming plastic in order to detect interactions and synergies with bacteria that have remained hidden.

*Galleria mellonella* is a cosmopolitan species (Kwadha et al., 2017) that has evolved in harsh habitats and developed the ability to digest variable diets during the larval stage, including honey, beeswax, and the skin of bee pupae (Jindra and Sehna, 1989). This ability suggests the combined action of gut bacteria and fungi in plastic degradation that may involve complicated interactions. The moth’s gut microbiota, though, did not have a clear role in this degradation when lab-reared larvae were examined (Kong et al., 2019). Given that the existence of a global resident gut microbiota has been questioned for several Lepidopteran species (Hammer et al., 2017), a first step to assess the importance of gut microbiomes in plastic biodegradation is to detect to what degree the gut bacteria and fungi of the Greater Wax Moth larvae consuming plastics change in comparison to natural diets. Assessments should include wax moth individuals from understudied regions of the world, where knowledge on the subject is limited, and unknown but key microorganisms may exist.

The aim of this study was to describe changes in the gut microbiome associated with the consumption of polyethylene and polystyrene by the Greater Wax Moth*, Galleria mellonella L.* (Lepidoptera, Pyralidae) in Argentina. Specifically, we aimed to: (1) analyze the composition, diversity, and structure of bacteria and fungi gut communities when the host was exposed to diets based on PE and PS, (2) identify biomarkers for further experiments on plastic degradation, and (3) identify associations between bacteria and fungi occurrence under these diets.

## Materials and Methods

### Microbial Communities’ Molecular Analyses

*Galleria mellonella* larvae were acquired at the CEMUBIO (Centro Multiplicador de Biocontroladores Nativos, National Institute of Agricultural Technology - INTA), in Río Negro, Argentina, where they were fed a sterilized artificial diet based on essential nutrients and proteins. The larvae were kept under this artificial diet until the experiments. Larvae with a clear color (i.e., healthy aspect) in the instars three to four were fed with polyethylene, polystyrene, and beeswax for 7 days, at 30°C and in absence of light. This time interval was determined based on our own previous trials in which longer intervals led to pupation and consequent loss of the digestive tubes. Similar feeding intervals have been used in previous studies to examine changes in gut microbiota in response to diet (e.g., Broderick et al., 2004; Ma et al., 2021). Four larvae were put per Petri dish with one 4 cm-side square of any of the three materials and the design was repeated five times for each diet (*n* = 20 per treatment). After 7 days, two larvae were randomly selected from each Petri dish and their body surfaces disinfected with alcohol 70% for 1 min. Then, the larvae were dissected to obtain their digestive tubes (*n* = 10 per treatment). The entire guts were used for analysis, including their contents. The 30 digestive tubes were individually placed in empty sterile Eppendorf’s at −80°C until DNA extraction.

Total DNA was extracted using the DNeasy Blood & Tissue Kit (QIAGEN) and at least 10 ng of the obtained DNA per larvae was quantified on a DeNovix DS-11 microvolume spectrophotometer (Supporting data and figures).^1^ This DNA was conserved in TAE buffer at −80°C and used for rDNA 16S amplification and sequencing.

### 16S-rRNA and ITS-2 Gene Amplifications and Read Processing

DNA from the 30 digestive tubes were normalized to 4 nm during l6S library prep. The V4 region of the 16S ribosomal RNA gene (~291 bp) was amplified using universal bacterial primers: 515F (5’GTGCCAGCMGCCGCGGTAA3’) and 806R (5’GGACTACHVGGGTWTCTAAT3’; Caporaso et al., 2012) and the ITS-2 gene was amplified with primers ITS9-FW and ITS4-RV with amplicon sizes ranging from 240 to 460 bp as described by de Souza et al. (2016). Raw read pre-processing of demultiplexed files was done with a Phred offset of 33, and default parameters (QIITA, Gonzalez et al., 2018). Reads were trimmed to 250 bp. 16S rRNA genes were classified using the SILVA database (Quast et al., 2013), while ITS reads were classified using the UNITE 7.0 taxonomic database (Nilsson et al., 2018) both with a minimum similarity threshold of 97%. OTU picking was performed using a closed reference approach. The species table (biom file) was downloaded from QIITA for downstream analyses using R and a locally run version of QIIME (Caporaso et al., 2010). Singletons (OTUs with less than three reads), sequences matching chloroplasts, mitochondria, those matching eukaryotes, and taxonomically unassigned sequences were removed from downstream analyses.

Rarefaction was done at a level of 95,035 reads for 16S rRNA and 95,043 reads for ITS (Table 1).

**Table 1:** Count average’s for sequences and features from 16S and ITS samples.

16S Sample Information		
Treatment Group	Sequence Count Average	Feature Count Average
Beeswax	27260.7 ± 4,580	417.8 ± 65
Expanded polystyrene	29527.1 ± 5,609	467 ± 19
Polyethylene	32,901 ± 4,413	475.5 ± 48
		
**ITS Sample Information**		
Treatment Group	Sequence Count Average	Feature Count Average
Beeswax	21527.2 ± 2,919	34.3 ± 5.7
Expanded polystyrene	19814.5 ± 2,121	29 ± 5.6
Polyethylene	16649.1 ± 2,407	28.4 ± 4.6

Reads are publicly available and deposited in QIITA project ID 12844 with its associated metadata, as well as in EBI accession number ERP135546.

## Community Analysis of Diversity

### Beta Diversity Measured as Bray–Curtis Dissimilarity

Quantification of compositional dissimilarity between sample groups was done using pairwise Bray-Curtis index (Bray and Curtis, 1957). Global differences in bacterial and fungal communities were visualized using Non-Metric Multidimensional Scaling (NMDS). Statistical significance between sample groups was assessed using the PERMANOVA test (Anderson, 2001). Additionally, permutational multivariate analysis of variance using distance matrices (adonis permutation test) between different groups (plastic treatments) with a calculated value of *p* based on the Bray-Curtis distance table which was used to generate the plots. These tests were done using the python script *compare_categories* for each specific test in QIIME with the distance matrix as the input file and 999 permutations.

### Alpha Diversity, Taxonomic Plots and Discriminant Taxa Analyses

Alpha diversity measures of Chao 1 (richness) and Shannon (diversity) were plotted as boxplots using *ggplot2* (Wickham, 2009; R Development Core Team, 2020). For alpha diversity statistical tests, we used nonparametric t-tests with Monte Carlo permutations to determine the value of *p*. Barplots revealing phyla and genus were computed using QIIME (Caporaso et al., 2010).

Biomarker discriminant taxa analyses were performed with the LEfSe algorithm (Segata et al., 2011) for multi-class comparison. In order to reduce false discovery rates, FDR values of *p* = <0.01 were considered for significant differences—these analyses were performed using the MicrobiomeAnalyst pipeline (Dhariwal et al., 2017).

OTU Ubiquity dot plots were built relating OTU relative abundances with OTU proportions among samples (ubiquity). The 16S rRNA and ITS OTU tables and the metadata files were modified into ubiquity matrices containing the OTU ubiquity (proportion of samples in which it is represented), and the OTU relative abundance, through the integration of multiple *R* programs such as *phyloseq* (McMurdie and Holmes, 2013), *vegan* (Oksanen et al., 2008), *reshape* (Wickham, 2007) and *phytools* (Revell, 2012). Dot plots were built with *ggplot2* (Wickham, 2009).

### Ubiquity and Co-occurrence Analysis Plots

To measure co-occurrence between fungi and bacteria across the different plastic treatments, we calculated the Dice index using previously described methods and scripts (Godoy-Vitorino et al., 2018). The Dice index measures similarity between samples (Dice, 1945), with values that range between 0 (no co-occurrence) to 1 (maximum co-occurrence).

## Results

During the experiments we noted some differences in the general condition of the larvae under the different diets. The most notorious was behavioral; the feeding activity of larvae in polyethylene and polystyrene-based diets was high during the first 2 days and almost absent in the last day, while larvae fed on beeswax showed a constantly intermediate feeding activity along the time interval.

### Structure and Diversity of Bacterial and Fungal Communities

**Figure 1:**
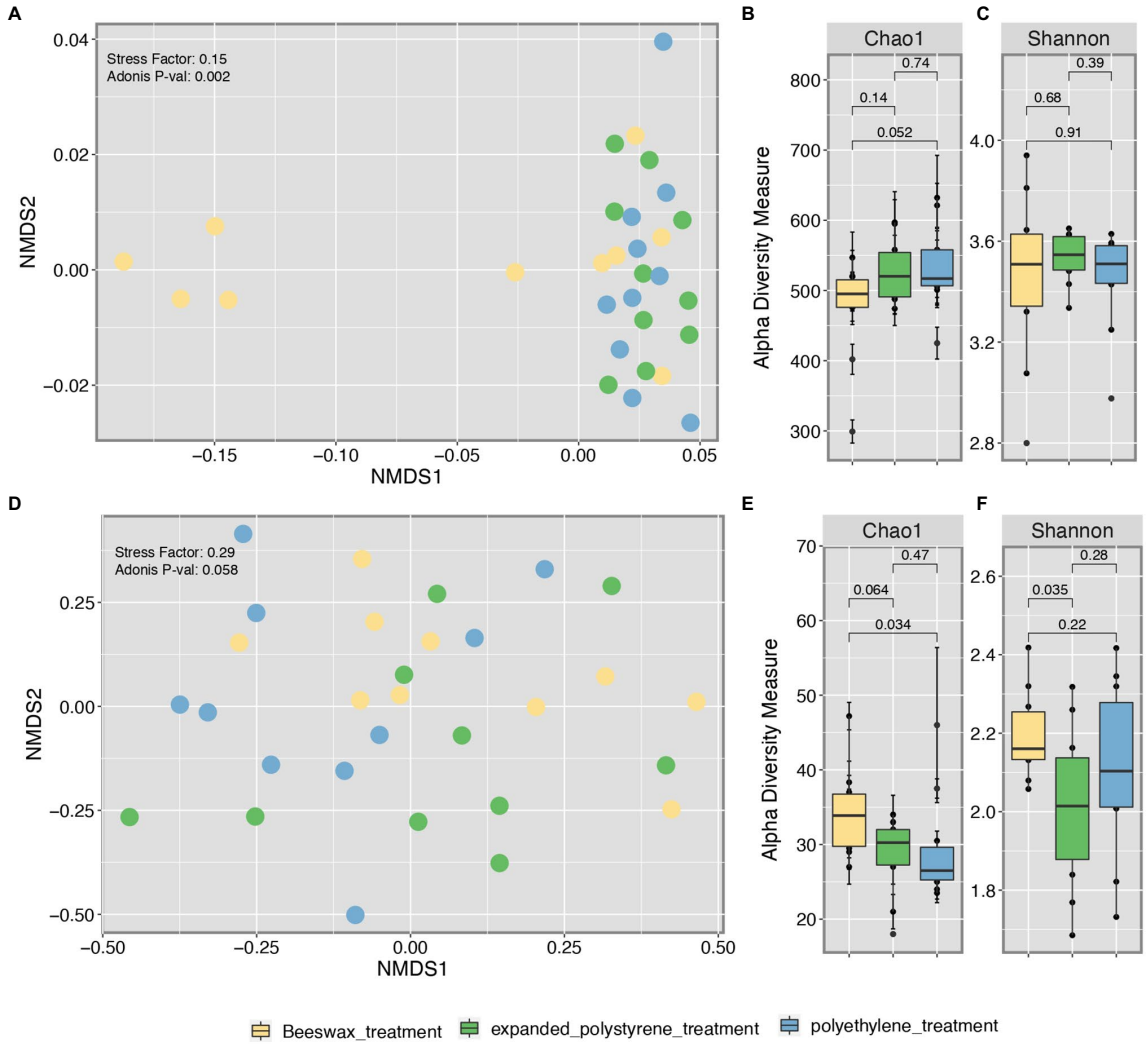
Beta diversity (NMDS) of bacterial **(A)** and fungal **(D)** communities. Species Richness based on Shannon **(B)** and Chao diversity indexes for bacterial **(B,C)** and fungal **(E,F)** communities with statistical significance value calculated through Wilcoxon pairwise test.

Beta diversity (i.e., structure) comparisons of gut bacterial communities, based on OTUs and using NMDS ordination, confirmed that there were differences in composition between bacterial communities in larvae fed with beeswax and communities in larvae fed with plastics (*p* = 0.02) as seen by the majority of beeswax samples to the left of Axis 1 (Figure 1A). In terms of the alpha diversity, the Chao richness index for the bacterial biota showed no differences among treatments, except for a slightly higher richness of gut bacteria in moths consuming polyethylene compared to beeswax (*p* = 0.052; Figure 1B), but no differences were found for Shannon diversity (Figure 1C).

As for the fungal microbiota, beta diversity comparisons of composition showed marginal to no differences among treatments (*p* = 0.058), with a majority of samples from plastic eaten moths to the left of Axis 1 (Figure 1D). However, in terms of richness (Chao1) and diversity (Shannon) we found significant differences, with the gut from beeswax consumers being significantly richer than those consuming polyethylene (*p* = 0.034) and marginally higher than expanded polystyrene (*p* = 0.064). Expanded polystyrene samples were the ones with the least diversity compared to beeswax (*p* = 0.035), and no differences were found in the fungal diversity among the plastic consumers (Figures 1E,F).

### Composition of Bacterial and Fungi Communities

**Figure 2:**
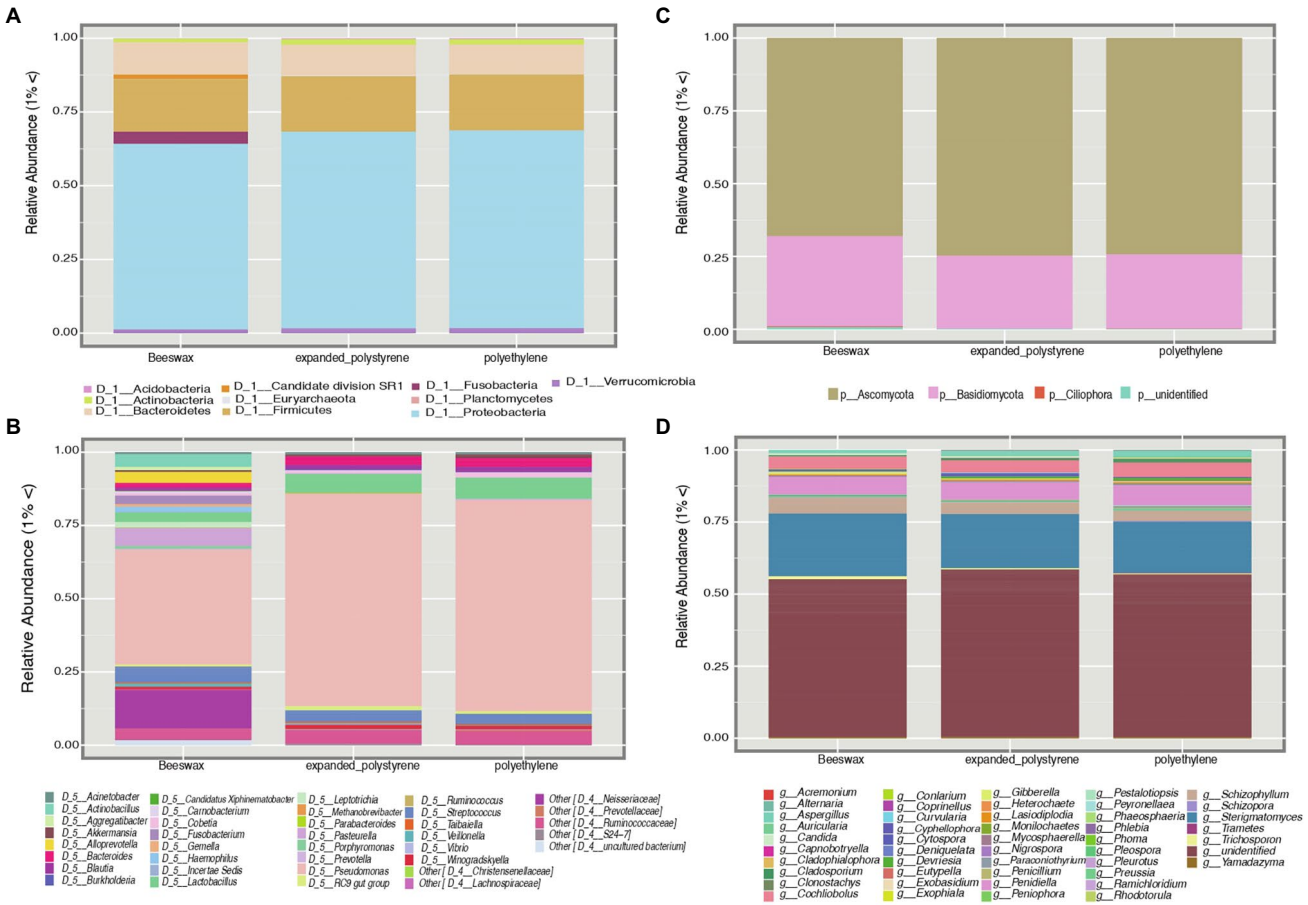
Composed panel depicting taxonomic plots of bacterial diversity at the phyla level **(A)** and genus level **(B)**. Plots corresponding to fungal diversity at the phyla **(C)** and genus level **(D)**. The legend shows the taxa with more than 1% of abundance.

**Figure 3:**
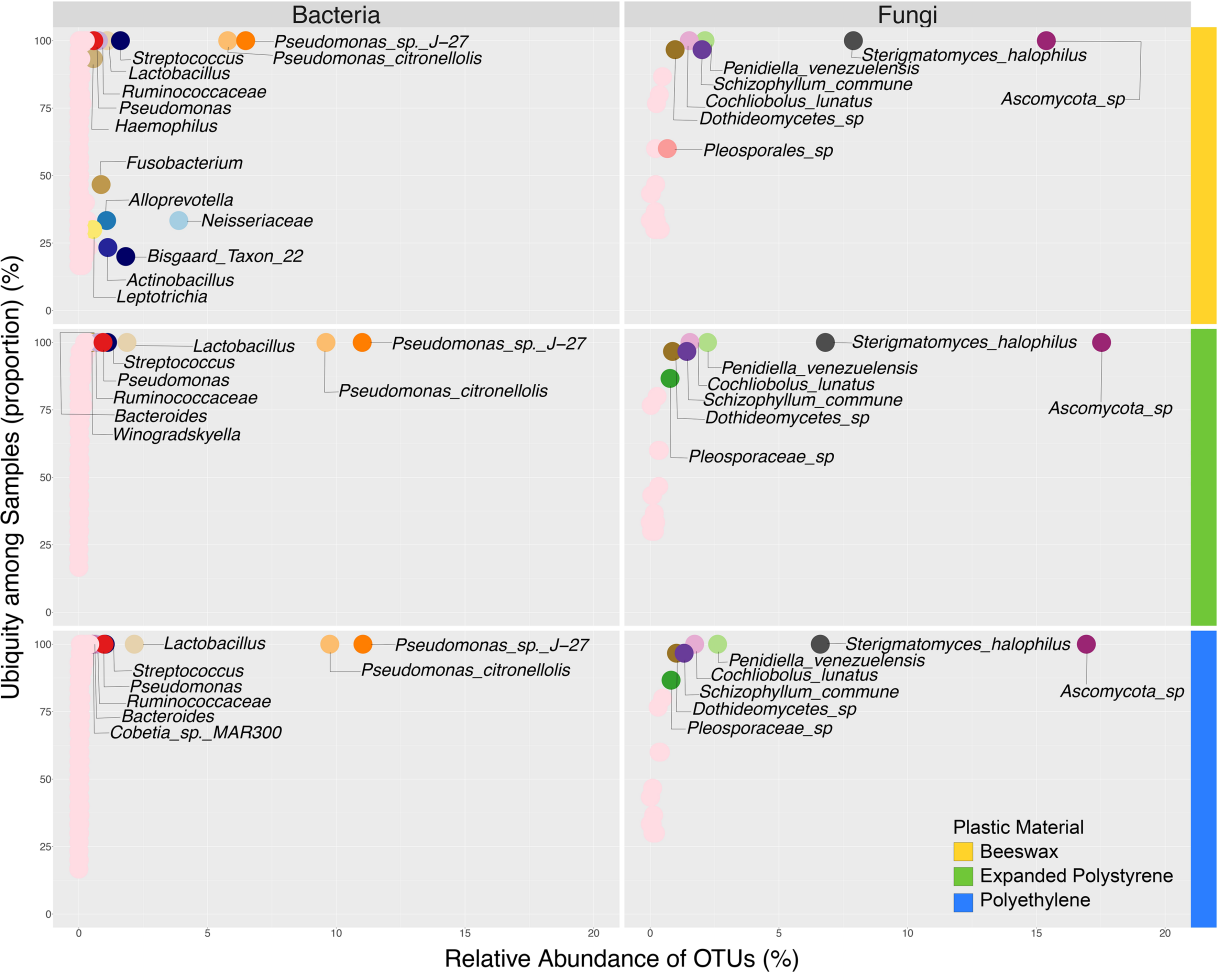
Ubiquity analysis of both bacterial and fungal biomes comparing relative abundance against prevalence in samples based on plastic material used for treatment.

At the bacterial phyla level, Proteobacteria were the most common across the three treatments, with a relative abundance of 72.2%, followed by Firmicutes (mean = 14.9%) and Bacteroidetes (mean = 7.9%; Figure 2A; Supplementary Table S1). Fusobacteria (mean = 1.5%) were found almost exclusively in the beeswax treatment, while Fibrobacteria (0.1%) was only present in the two plastic treatments (Figure 2A). At the genus level, *Pseudomonas* and *Lactobacillus* were the most common, representing, on average, 56.5 and 5.2%, respectively (Figure 2B; Supplementary Table S2). These genera were found in a lower proportion in the beeswax diet in contrast to the plastic treatments; as per the ubiquity plot (Figure 3), the relative abundance of *Pseudomonas* sp., and *P Citronellolis* increased from a ~ 5 to 10% in plastic consumers. Members of Neisseriaceae (mean 3.8% of the total), *Pasteurella* (1.8%), *Actinobacillus* (1.3%), *Alloprevotella* (1.1%), *Leptotrichia* (0.5%), *Aggregatibacter* (0.4%), *Porphyromonas* (0.3%), *Gemella* (0.3%), *Carnobacterium* (0.2%), *Donghicola* (0.1%), *Eikenella* (0.1%) were present in the beeswax diet and absent in plastic treatments. In contrast, *Bifidobacterium, Butyricimonas, Alistipes, Fibrobacter, Marinococcus, Beijerinckiaceae, Bradyrhizobium, Rhizobium, Roseovarius, Sphingomonas, Enhydrobacter* were found in the plastic treatments but not in beeswax and in a proportion of 0.1% (Figure 2B).

Considering fungi, at the phylum level only two groups were found, Ascomycota (mean = 72.1%) and Basidiomycota (mean = 27.9%; Figure 2C; Supplementary Table S3). Ascomycota occurred above the average in plastic treatments, while Basidiomycota was present above average in the beeswax treatment. At the genus level, an unidentified Ascomycota group was, on average, the most common (49.6%), the second most represented genus was *Sterigmatomyces* (mean = 21.4%) followed by *Penidiella* (mean = 7%). These genera were found in all three treatments (Figures 2D, 3). Some genera with low representation (< 5%), such as *Schizophyllum* and *Trichosporon*, although present in all three treatments, were more common in the beeswax diet. An undefined group of Capnodiales (Ascomycota), showed percentages <0.1%, and were observed only in plastic treatments.

### Biomarker Tests

**Figure 4:**
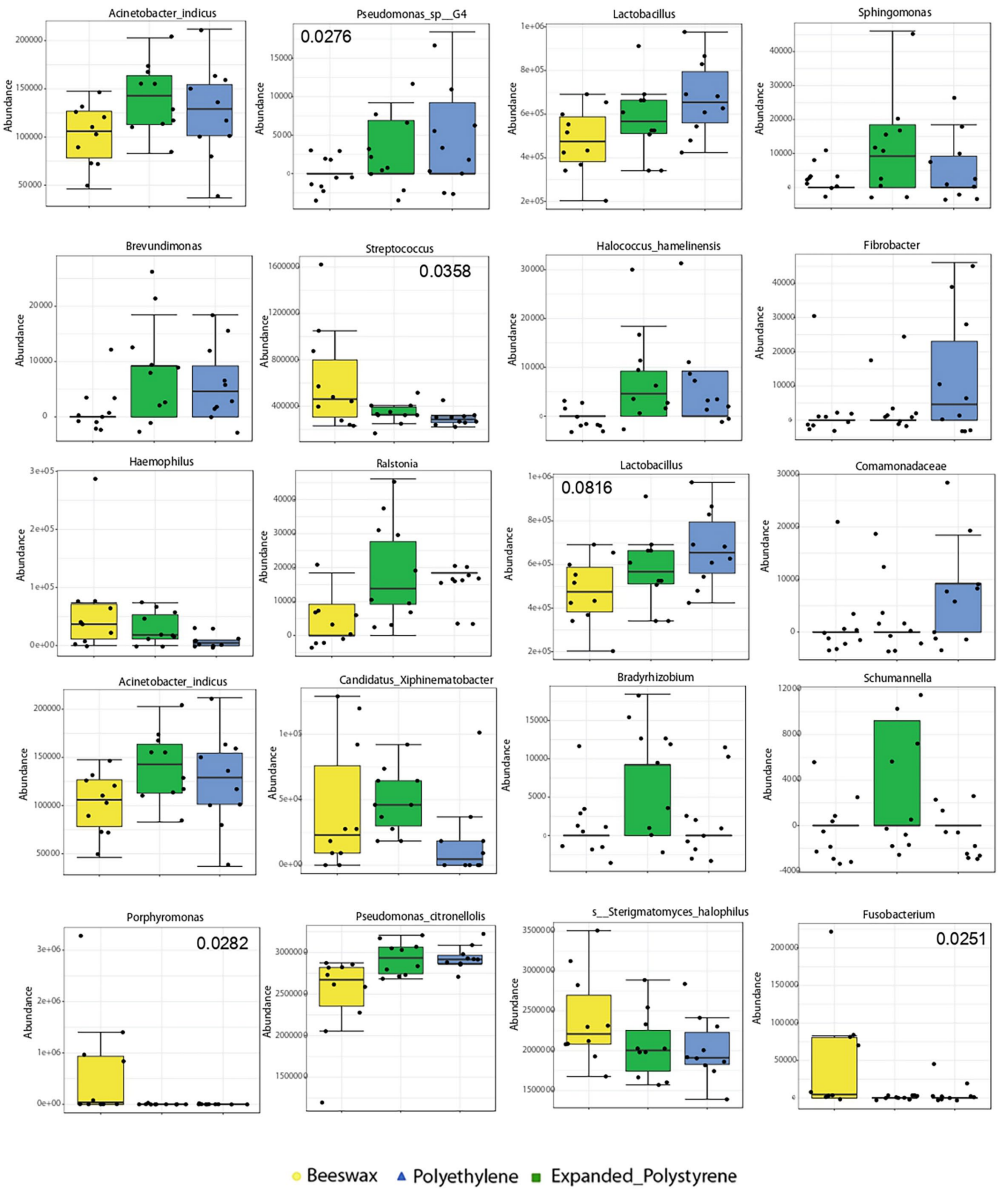
Biomarker discriminant taxa analyses of bacterial communities according to the treatment, *via* LEfSe (Linear discriminant analysis Effect Size) algorithm, including only OTUs present in at least 50% of the samples. Values of *p* are in some taxa added to the plot.

Bacterial taxa that best characterized each treatment, and changed according to the larval diet are shown in Figure 4. *Pseudomonas* sp. (*p* = 0.0276), *Pseudomonas citronellolis* and *Lactobacillus* (*p* = 0.0816) were significantly more abundant in the plastic treatments compared to beeswax.

Among Proteobacteria, the Comamonadaceae family as well as the genera *Pseudomonas* sp. G4*, Sphingomonas, Halococcus, Brevundimonas* and *Bradhyrhizobium* were present in plastic-fed larvae. Other bacterial groups inhabiting the guts of plastic-fed larvae were found, such as members of *Schumannella* belonging to the phylum Actinobacteria and populations of the genus *Fibrobacter* belonging to the Fibrobacteres phylum. The genus *Schumannela* was exclusively related to a diet based on polystyrene and *Fibrobacter* was exclusively related to a diet based on polyethylene. On the contrary, the phylum Fusobacteria was exclusively found in beeswax-fed larvae (*p* = 0.0251). Additionally, *Streptococcus* (*p* = 0.0358), *Porphyromonas* (*p* = 0.0282) were dominant in beeswax (Figure 4).

**Figure 5:**
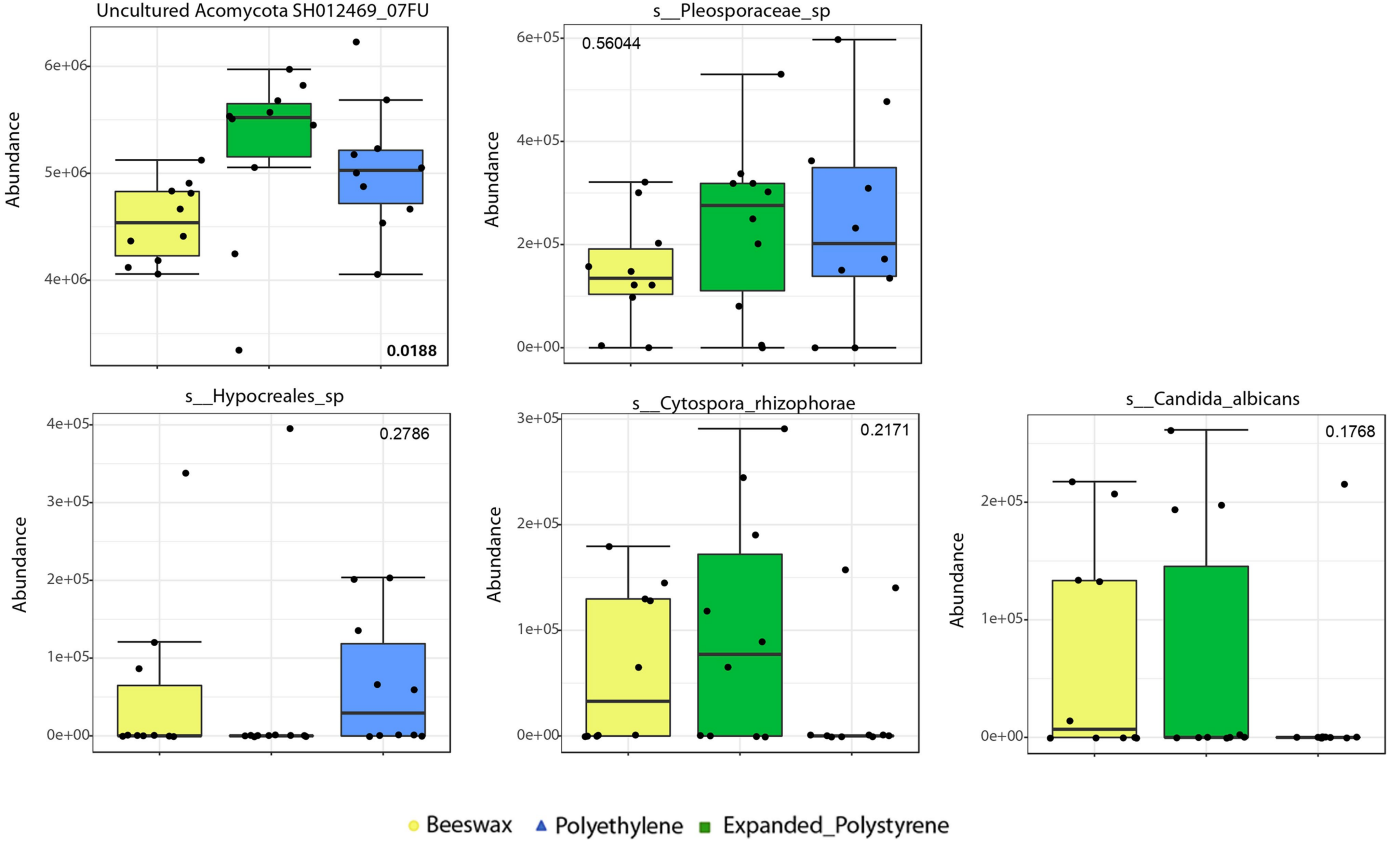
Biomarker discriminant taxa analyses of fungal communities according to the treatment, *via* LEfSe, including only OTUs present in at least 50% of the samples. Taxa shown here correspond to genus level or to the lowest unclassified taxa. values of *p* are in some taxa added to the plot.

Results of the biomarker discriminant taxa analyses (LEfSe) of relevant fungal groups are shown in Figure 5. Analysis showed that polystyrene consumers had a high prevalence of Ascomycota (*p* = 0.0188) as dominant taxa.

### Co-occurrence Analysis

**Figure 6:**
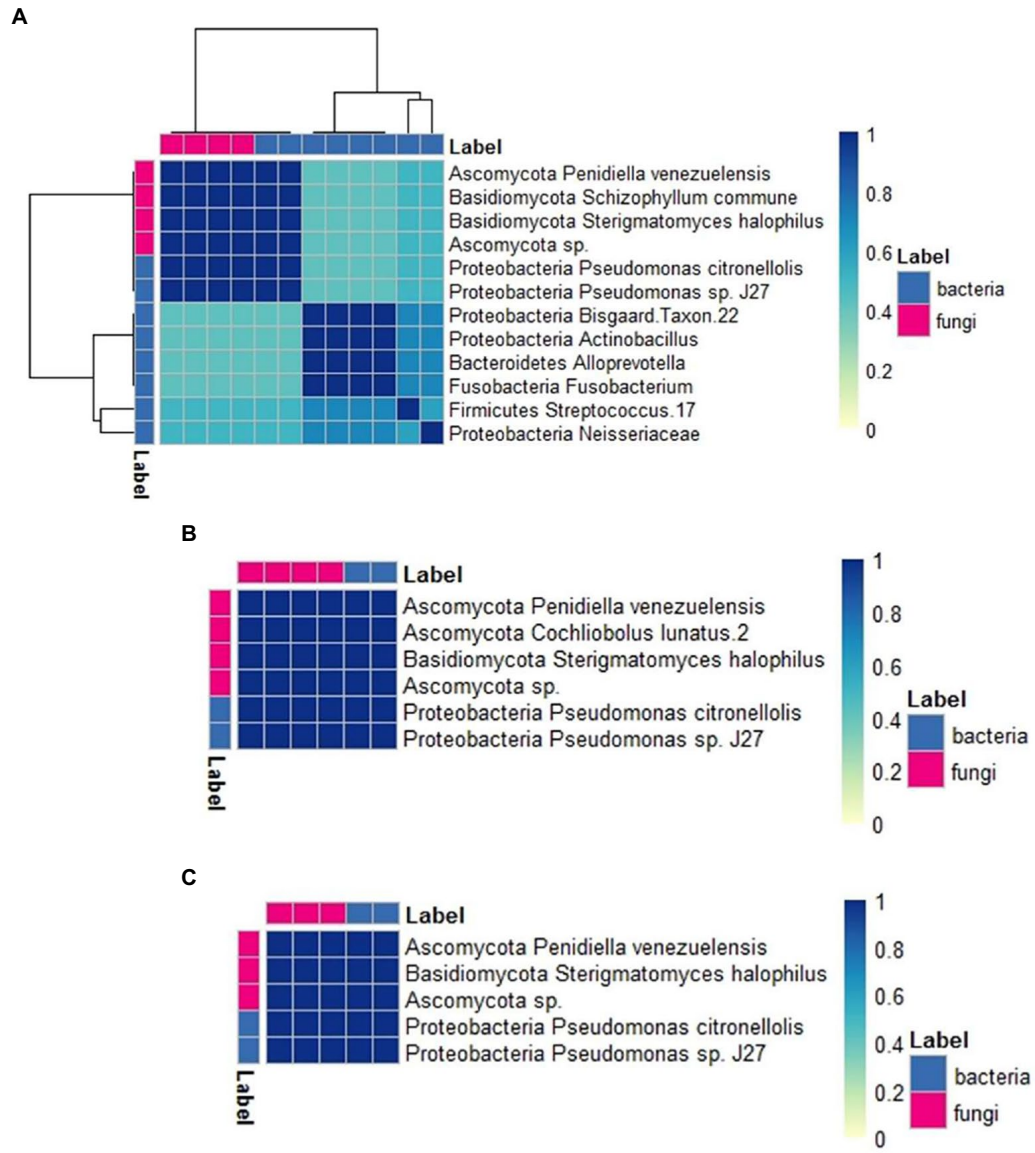
Co-occurrence analysis heat map showing the degree of association between bacteria and fungi taxa across diets, scored using the Dice index. Clustering obtained from Euclidean distances between taxa. **(A)**: beeswax, **(B)**: polyethylene, **(C)**: expanded polystyrene.

The larval diets influenced the co-occurrence of species composing of the wax moth gut microbiota. The associations between bacteria and fungi were more diverse in the beeswax diet (Figure 6A), with a number of bacterial and fungal taxa disappearing in the plastic treatments, such as members of Neisseriaceae, *Streptococcus*, *Fusobacterium*, *Alloprevotella*, *Actinobacillus*, an uncultured Proteobacteria, and *Schizophylum commune* (Basidiomycota) (Figures 6B C). Nevertheless, we found a group of both bacteria and fungi that co-occurred in all diets: *Pseudomonas* sp. J27 and *P. citronellolis*, Ascomycota sp., *Penidiella venezuelensis* (Ascomycota) and *Sterigmatomyces halophilus* (Basidiomycota). These five OTUs co-occurred in all treatments and individuals. Between plastics, the polyethylene treatment included a fungal taxon that was absent in the expanded polystyrene diet, *Cochliobolus lunatus* (Ascomycota).

## Discussion

This is the first multi-kingdom description of the Greater Wax Moth’s gut microbiome associated with the consumption of polyethylene and polystyrene. Gut bacterial and fungal communities of wax moth larvae in Argentina responded differently to the various diets. There were differences in structure and composition between bacterial communities in larvae consuming beeswax and bacterial communities in larvae consuming PE and PS, while fungal communities showed differences mostly in richness and diversity. In addition, we identified both bacterial and fungal biomarkers that may be useful in future experiments and identified co-occurrence of bacteria and fungi that suggest further research avenues.

The response of wax moth gut microbiota to diet was different in bacteria and fungi. Bacterial composition differed between beeswax- and plastic-based diets; particularly, a dominance of *Streptococcus, Porphyromonas* or *Fusobacteria* was observed in the beeswax diet. This result agrees with previous reports on *G. mellonella* and other insects’ gut communities of individuals consuming plastics (Przemieniecki et al., 2019; Cassone et al., 2020) and suggests that plastics, a low-quality resource, changes gut bacterial communities. A possible simplification of communities caused by a plastic-based diet may have ecological consequences for the larvae. Alpha diversity, in contrast, was not different among bacterial communities under the three diets. An opposite result has been reported by Cassone et al. (2020) in Canada, which highlights the importance of screening individuals from different geographic locations to understand gut microbiota-plastic interactions. In comparison to bacteria, fungal communities did not differ in composition among diets however significant differences were found in richness and diversity. This suggests that bacteria communities were more sensitive to changes in diet than fungal communities—only changing in fungal relative abundance—in our study, which in turn may suggest a more active role of bacteria in plastic decomposition, in comparison to fungi.

In our study, abundance of different *Pseudomonas* populations (Pseudomonadales), *Bradyrhizobium* (Rhizobiales) and Comamonadaceae (Burkholderiales) populations in the gut of plastic-fed larvae was noticeable. According to other studies, most bacteria that enter into symbiotic relationships with insects belong to the orders Burkholderiales, Pseudomonadales, Rhizobiales, Xanthomonadales (Proteobacteria phylum) and Verrucomicrobiales (Verrucomicrobia phylum; Russell et al., 2009; Sapountzis et al., 2015). In contrast to our study, in which we found Comamonadaceae associated with a polyethylene diet in wax moths, Peydaei et al. (2021) reported this association in polystyrene fed wax moths. In accordance to our results, Cassone et al. (2020) found a dominance of the Proteobacteria phylum in wax moth larvae consuming plastics; but the genera they identified were different from those in this study, except for *Pseudomonas*. Species of the genus *Pseudomonas* are among the most cited degraders for a wide range of plastics (Espinosa et al., 2020; Cf et al., 2021); they have also been touted for the bioremediation of crude oil, simple hydrocarbons, naphthalene, toluene and other hydrophobic polymers (Wilkes and Aristilde, 2017). The extent to which plastics are biodegraded depends on both the structural arrangement of the polymer and the type of *Pseudomonas* strain. Consequently, to isolate new and more degrading-efficient *Pseudomonas* strains continues to be the focus of worldwide screening programs. Sphingomonas, detected in our study, are also well-known degraders (Coppotelli et al., 2010). At the same time, populations of the nitrogen-fixing bacteria *Bradyrhizobium* were certainly the most biologically informative group associated with the polystyrene diet. This bacterium could promote indirect re-assimilation of NH3 and atmospheric nitrogen to promote insect survival in nitrogen-deficient environments. Previous evidence on insects other than *G. mellonella* demonstrated that Proteobacteria and selected species of Actinobacteria supply insects with food by the production of amino acids and are predominant in the digestive tract (Hongoh et al., 2005, 2008; Desai and Brune, 2012). Proteobacteria which were exclusively related to polyethylene-diet such as *Fibrobacter* in our study have been major bacterial degraders of lignocellulosic biomass in the herbivore gut. Novel lineages are also present in other anoxic environments where cellulose degradation occurs, such as termite gut (Cragg et al., 2015). Considering that there is a growing interest in degradation of plastics materials using microorganisms capable of degrading lignin, starch, and cellulose (Mehmood et al., 2016), the presence of *Fibrobacter* in the gut of larvae fed polyethylene seems quite outstanding. The role of microbial hydrolase and oxidoreductase found in lignocellulosic degraders is well-known and is potentially useful for plastics degradation (Danso et al., 2019). The predominance of *Fibrobacter* sp. as well as *Pseudomonas* strains in the gut of larvae fed polyethylene and/or polystyrene could indicate that these bacteria have mechanisms to break down plastic polymers that are difficult to decompose.

The biomarker discriminant taxa analysis allowed detecting a major difference in predominant bacterial groups according to the treatment. The microbiota found in beeswax-fed larvae differed most considerably from the remaining bacterial groups since Fusobacteria were exclusively associated with this diet. *Fusobacterium* species are considered opportunistic pathogens in humans and other animals in which they compose the normal microbiota in the gastrointestinal tracts (Spector, 2009). Populations of Fusobacteria were found inhabiting the intestinal tract of *T. molitor* infected and uninfected with *Hymenolepis diminuta* (Fredensborg et al., 2020) as well as in the gut of the brown planthopper (Ojha and Zhang, 2019).

In relation to fungi, major changes in gut community composition under different diets were not observed in our study; in general, we found significant differences in richness and diversity (relative abundance), mostly Ascomycota characterized the guts of plastic-fed larvae, while Basidiomycota characterized the guts of beeswax-fed larvae. Common symbiotic gut fungi in insects are trichomycetes *sensu lato* (lineages within the Zygomycota; Cafaro, 2002; Valle and Stoianova, 2020), yeasts (Ascomycota; Malassigné et al., 2021), and Basidiomycota (Grigorescu et al., 2018). The genera we found in the guts of larvae in all three treatments were an unidentified Ascomycota group, followed by *Sterigmatomyces* (Basidiomycota), and *Penidiella* (Ascomycota), respectively. In accordance with our results, Zhang et al. (2020) identified Ascomycota in the guts of wax moth larvae consuming plastics, though *Aspergillus flavus* was not detected in our study. An undefined group of Capnodiales was present in low abundances only in the plastic treatments and this group differs from that of *Aspergillus* (Eurotiales). Species found in our study are not frequently reported in the literature regarding mechanical destruction with possible subsequent decomposition by microorganisms (Cf et al., 2021). Similarly to bacterial communities’ patterns, fungal species may differ geographically; but, we suggest that species that were present in all three treatments should be taken as candidate species when describing the core microbiota of *G. mellonella*.

Our co-occurrence analysis suggested associations between bacteria and fungi and their response to different diets in *G. mellonella* larvae for the first time. This approach takes into account synergic effects that could arise from the joint metabolic activities of bacteria and fungi in relation to the environment (e.g., Cucini et al., 2020). It has been previously used to describe multi-kingdom ensembles and their behavior under different conditions, in insects of economic importance (Guo et al., 2020), in ruminants (Pitta et al., 2016), and in humans (Godoy-Vitorino et al., 2018). In our study, it helped to identify five taxa that co-occurred in all treatments and individuals, and thus may be participating in core processes. This study also helped to evaluate the hypothesis that states that the dominance of some bacteria in gut communities may facilitate the entrance of entomopathogenic fungi (e.g., Gichuhi et al., 2020), which may have entered from the environment as a result of a depression of the immunological system driven by a low-quality diet such as plastics (e.g., Unckless et al., 2015). But this pattern was not supported by our data. The interactions between bacteria and fungi might play a key role in the plastic biodegradation process, and we showed a first step in identifying these associations using larvae in Argentina.

Our study is the first one to simultaneously identify bacterial and fungal communities associated with the gut of the Greater Wax Moth larvae fed with the two most common plastics: polyethylene (PE) and polystyrene (PS). In this sense, our results are promising in that it sheds light on possible alternatives of plastic pollution mitigation. However, the overproduction of plastic, especially single-use plastics, and its excessive discarding highlight the urgent need for responsible consumption by the entire society.

## Data Availability Statement

Reads are publicly available and deposited in QIITA project ID 12844 with its associated metadata, as well as in EBI accession number ERP135546.

## Author Contributions

JRB, EM, AG-C, AM, RC, AC, ACM-G, and FG-V contributed to the study conception and design. Material preparation was performed by JRB, EM, AG-C, MO, RC, AC, and ACM-G. Data collection and DNA sequencing were performed by BV-C and FG-V. Data analysis was performed by JRB, BV-C, EM, AG-C, AA, AM, MO, RC, ACM-G, and FG-V. The first draft of the manuscript was written by all authors. Financial resources were searched by EM, AG-C, AM, RC, AC, ACM-G, and FG-V. All authors contributed to the article and approved the submitted version.

## Funding

Support for DNA sequencing and publication fees was provided by the PR-INBRE program BiRC core (NIH/NIGMS award number P20 GM103475), Agencia Nacional de Promoción Científica y Tecnológica (PICT2017-Ð833) from Argentina supported larvae acquisition, diet experiments, and DNA extraction costs. Secretaria de Estado e Innovación y Desarrollo Tecnológico from Tucumán, Argentina supported the costs of experiments.

## Conflict of Interest

The authors declare that the research was conducted in the absence of any commercial or financial relationships that could be construed as a potential conflict of interest.

## Publisher’s Note

All claims expressed in this article are solely those of the authors and do not necessarily represent those of their affiliated organizations, or those of the publisher, the editors and the reviewers. Any product that may be evaluated in this article, or claim that may be made by its manufacturer, is not guaranteed or endorsed by the publisher.

## Footnotes

^1^ https://www.qiagen.com/zh-us/products/discovery-and-translational-research/dna-rna-purification/dna-purification/genomic-dna/dneasy-blood-and-tissue-kit/
